# Tumour Necrosis Factor Superfamily Members in the Pathogenesis of Inflammatory Bowel Disease

**DOI:** 10.1155/2014/325129

**Published:** 2014-06-17

**Authors:** Tomasz J. Ślebioda, Zbigniew Kmieć

**Affiliations:** Department of Histology, Medical University of Gdańsk, Dębinki 1, 80-211 Gdańsk, Poland

## Abstract

Inflammatory bowel disease (IBD) is a group of inflammatory conditions of the gastrointestinal tract of unclear aetiology of which two major forms are Crohn's disease (CD) and ulcerative colitis (UC). CD and UC are immunologically distinct, although they both result from hyperactivation of proinflammatory pathways in intestines and disruption of intestinal epithelial barrier. Members of the tumour necrosis factor superfamily (TNFSF) are molecules of broad spectrum of activity, including direct disruption of intestinal epithelial barrier integrity and costimulation of proinflammatory functions of lymphocytes. Tumour necrosis factor (TNF) has a well-established pathological role in IBD which also serves as a target in IBD treatment. In this review we discuss the role of TNF and other TNFSF members, notably, TL1A, FasL, LIGHT, TRAIL, and TWEAK, in the pathogenesis of IBD.

## 1. Introduction

Inflammatory bowel disease (IBD) is a group of inflammatory conditions of the gastrointestinal (GI) tract. Its two major forms are ulcerative colitis (UC) and Crohn's disease (CD). Crohn's disease affects mainly small intestine and colon, although any other segment of the GI tract may also be involved. CD is characterized by discontinuous ulcerations and bowel wall inflammation. UC manifests by inflammation of the colon mucosa that in most cases extends to the rectum. Typical symptoms of IBD are abdominal pain, diarrhoea, and rectal bleeding as well as weight loss, fever, and fatigue. Furthermore, CD patients often develop strictures between segments of the bowel or between the bowel and other organs [1]. IBD is an autoimmune disorder of unknown aetiology that results from excessive immune responses to intestinal microbiota which are triggered by increased activity of effector T cells and/or decreased activity of regulatory T cells, changes in the composition of intestinal microflora, and/or damaged epithelial barrier [1, 2]. Recently, Hand et al. [3] showed in a mouse model that acute infection of the GI tract results in the loss of CD4(+) T cell tolerance of commensal antigens and priming of adaptive immune response directed against commensal bacteria which contributes to the development of IBD. Furthermore, 5–16% of IBD patients report a family history of the disease [4] which indicates that it may be associated also with a genetic background. Indeed, there are several genetic factors that contribute to the pathogenesis of the IBD which include genetic mutations leading to enhanced inflammatory response [5–7], defective elimination of intracellular bacteria [8, 9], or disruption of the intestinal epithelial barrier [10]. There are also certain environmental risk factors for IBD that include (1) treatment with nonsteroidal anti-inflammatory drugs which damage intestinal mucosa, making it more permeable to bacteria; (2) taking oral contraceptives that elevate the level of estrogens which act as enhancers of humoral immunity; (3) smoking that increases risk of acquiring CD, although it appears to play a protective role in UC through yet unknown mechanisms; and (4) limitation of exposure to enteric pathogens in childhood due to antibiotic treatment or living in hygienic environment [2]. Association of IBD with other environmental factors such as diet rich in sugars and fats and living in urban environment or stress remains currently controversial [2].

Considering the type of immune response, IBD is not a uniform disease; in CD the inflammation is mainly driven by T helper 1 (Th1) or T helper 17 (Th17) cells, while UC is considered to be generally a T helper 2- (Th2-) mediated condition [11]. It has to be noted, however, that the strict polarization model of Th1, Th2, and Th17 is not fully applicable in IBD due to a redundancy of effector and regulatory pathways affected by factors such as the phase of the disease (remission or acute bouts), innate inflammatory mechanisms, or anti-inflammatory treatment of patients [12]. For example, during the remission phase of the disease, the level of a Th2 cytokine, interleukin 13 (IL-13), is higher in peripheral blood mononuclear cells (PBMCs) isolated from patients with CD than in PBMCs isolated from patients with UC [13]. Other reports show that the frequency of Th1 (IFN-*γ*(+) CD4(+)) T cells is lower in the peripheral blood of paediatric IBD patients than in healthy control subjects [14, 15] and it increases with patients' age [15]. Furthermore, the cytokine expression profile in IBD patients does not usually reflect fully differentiated Th1, Th2, or Th17 immune responses [12]. In UC, expression of a typical Th2 cytokine, IL-4, was not elevated in intestinal mucosa of UC patients [12]. Instead, it has been suggested that the central role in the pathogenesis of UC is played by IL-13 [12, 16] which not only acts as a Th2 effector cytokine [17] but also disrupts the continuity of colonic epithelium by inducing apoptosis of epithelial cells and upregulating expression of claudin-2, a pore-forming tight junction protein [18]. To add more complexity to the pathogenesis of IBD, a recent report by Mannon et al. [19] has showed that in some patients UC is characterized by elevated production of a Th17-specific cytokine, IL-17A, by intestinal lamina propria T cells.

## 2. Tumour Necrosis Factor Superfamily 

There are 19 ligands and 29 receptors identified to date that constitute the tumour necrosis factor superfamily (TNFSF) [20]. Their expression pattern and structural attributes allow them to activate signalling pathways that lead to cell survival, proliferation, differentiation, or apoptosis. TNFSF receptors can be divided into two groups depending on the presence or absence of the intracellular death domain (DD). Signalling via the death domain requires the participation of adaptor proteins FADD (Fas-associated death domain) and TRADD (TNF receptor-associated death domain) and leads to activation of caspases which typically results in apoptotic death of a cell [21]. The second group of TNFSF receptors signals only via adaptor proteins termed TRAFs (tumour necrosis factor receptor-associated proteins), although DD-containing receptors can also utilize this pathway. TRAFs bind either to TRADD or directly to the cytoplasmic part of the receptor and initiate signal transduction pathways that lead to the activation of several transcription factors, such as AP-1 and NF-*κ*B, responsible for the activation of prosurvival genes [21], although they are involved also in proapoptotic signalling [22–24]. Hence, functional activity of TNFSF receptors largely depends on the cellular context and the balance between pro- and antiapoptotic factors inside the cell and in the environment.

Most TNFSF members are expressed on cells of the immune system and play an important role in maintaining the equilibrium of T cell-mediated immune responses by providing direct signals required for full activation of effector and regulatory T cells, regulation of their expansion, contraction of the T cell effector pool, and survival of memory T cells [25–30]. For these reasons, members of the TNFSF are involved in the pathogenesis of many T cell-mediated autoimmune diseases, such as asthma, diabetes, or arthritis [26]. Many recent reports indicate that certain TNFSF members, notably, TNF (tumour necrosis factor, TNFSF2, also known as TNF-*α*) [31], TL1A (TNF-like protein 1A, TNFSF15) [32, 33], FasL (TNFSF6) [34–36], LIGHT (lymphotoxin-like inducible protein that competes with glycoprotein D for binding herpesvirus entry mediator on T cell, TNFSF14) [37], TRAIL (TNF-related apoptosis inducing ligand, TNFSF10) [38], and TWEAK (TNF-like weak inducer of apoptosis, TNFSF12) [39], contribute to the pathogenesis of IBD not only by enhancing proinflammatory function of T cells but also by direct disruption of the integrity of intestinal epithelium (Table 1).

## 3. TNF 

Tumour necrosis factor (TNF; TNF-*α*; TNFSF2) is biologically active in the form of homotrimeric transmembrane or soluble protein [40]. It is expressed by macrophages, T cells, B cells, NK cells, mast cells, endothelial cells, fibroblasts, and neurons; its expression is strongly upregulated by certain proinflammatory factors such as lipopolysaccharide (LPS) or other bacterial products and IL-1*β* [20, 24, 41]. There are two types of TNF receptors, the death domain-containing TNFR1 (TNF receptor 1, also known as p55 or TNFRSF1A), which is constitutively expressed on most nucleated mammalian cells and is activated by both the transmembrane and soluble form of TNF [20], and TNFR2 (TNF receptor 2, also known as p75 or TNFRSF1B) which does not contain the death domain and is activated only by the transmembrane form of TNF [24]. Expression of TNFR2 is strictly regulated and found mostly on certain populations of lymphocytes, including T-regulatory cells (Tregs), endothelial cells, microglia, neuron subtypes, oligodendrocytes, cardiac myocytes, thymocytes, and human mesenchymal stem cells [20, 42].

Elevated expression of TNF was detected in IBD patients more than 20 years ago [52]. The level of TNF mRNA was upregulated in involved colonic tissue of CD patients [53] as well as in both involved and uninvolved colonic tissue of UC patients [54] compared to healthy subjects. A recent report [55] showed that elevated concentration of TNF protein that correlated with the activity of the disease was present in blood serum of CD patients while other groups [52, 56] found increased levels of TNF protein both in serum [52, 56] and in the intestinal lamina propria of both CD and UC patients as well as the intestinal submucosa of CD patients [57]. The production of TNF in the colon mucosa of UC patients was localized to lamina propria macrophages [57]. Although several groups did not detect increased levels of TNF protein or mRNA in blood serum or colon mucosa of IBD patients, respectively [58, 59], successful use of anti-TNF agents in IBD therapy [31] documents that TNF belongs to the major effector molecules involved in the pathogenesis of CD and UC. It is worth to note, however, that a recent study on a mouse model of T cell-mediated colitis has shown that only neutralization of the transmembrane, but not soluble, TNF form induced remission of experimental colitis [60]. Pathogenesis of IBD is associated also with altered expression of TNF receptors since both CD and UC patients showed elevated expression of TNFR2 on colonic epithelial cells [61]. Moreover, a positive correlation was observed between CD and UC activity and serum concentration of soluble forms of TNFR1 and TNFR2 [55]. Furthermore, upregulated expression of TNFR2 (but not TNFR1) was found on intestinal lamina propria CD4^+^ cells as well as peripheral blood T cells of CD patients [62].

### 3.1. Role of TNF in the Dysregulation of Intestinal Barrier Permeability

Several studies showed that TNF contributes to the disruption of intestinal epithelial barrier which allows for intestinal penetration of luminal antigens and promotes intestinal inflammation (Table 2) [63–65]. Intestinal epithelium integrity is provided by the presence of tight junctions (TJ) located in the apical region of intestinal epithelial cells. Data obtained* in vitro *by Ma et al. [63] showed that stimulation of colonic epithelial Caco-2 cells with TNF downregulated the expression of TJ-associated zonula occludens-1 proteins and altered their junctional localization in an NF-*κ*B-dependent manner.

Transmembrane expression of TNF is regulated by a pleiotropic metalloproteinase ADAM17 which is involved in the cleavage of transmembrane TNF and its shedding from the cell surface [66]. Cesaro et al. [67] reported early posttranscriptional upregulation of ADAM17 in intestinal mucosa of patients with highly active CD and, in an* in vitro* model, in intestinal epithelial cells, which was linked to transepithelial migration of polymorphonuclear neutrophils. Treatment of TIMP3-deficient colonic epithelial cell line HT29-C1.16E with TIMP3, an inhibitor of ADAM17 activity, decreased TNF shedding and sensitized the cells to TNF-mediated epithelial hyperpermeability due to the downregulation of zonula occludens-1 proteins [64]. Other reports showed that IBD patients had also elevated mucosal expression of another TNF sheddase, metalloprotease ADAM19, localized mainly in epithelial cells [68], whereas a mouse study demonstrated that shedding of TNF can be mediated also by matrix metalloproteinase 13 (MMP13) [69].

Epithelial barrier dysfunction can be mediated also by increased expression of myosin light chain kinase (MLCK) followed by subsequent phosphorylation of myosin II regulatory light chain (MLC) which results in the contraction of the perijunctional ring composed of actin and myosin filaments [73]. Expression of MLCK was elevated in ileal and colonic epithelium of CD and UC patients and correlated with the activity of the disease [74].* In vitro *investigation showed that TNF upregulated expression of MLCK in Caco-2 cells pretreated with IFN-*γ* which increased expression of TNF receptors on the cell surface [65]. A recent study on TNFR1 or TNFR2-deficient mice showed that upregulation of MLCK and the loss of intestinal epithelial barrier in CD4(+) CD45RB (high) T cell transfer model of intestinal inflammation were dependent on TNFR2 expressed on intestinal epithelium but not TNFR1 [70].

In an elegant* in vivo* study on mouse models, Marchiando et al. [71] showed that TNF induced redistribution of several TJ and adherens junction proteins, including zonula occludens-1, occludins, claudins, and E-cadherin, as well as MCLK, to basolateral membranes of intestinal epithelial cells. Furthermore, administration of TNF resulted not only in the rearrangement of junctional proteins but also in the shedding of whole cells from intestinal epithelium. These events were preceded by caspase-3 activation due to the TNF-induced activation of NF-*κ*B-dependent signalling pathway and of proapoptotic pathways [71]. These data suggest that TNF-triggered loss of intestinal epithelium integrity is a complex process which involves not only rearrangement of cytoskeletal elements but also direct induction of intestinal cells' apoptosis by TNF. Indeed, studies on mice showed TNF-induced apoptosis of intestinal epithelial cells in a TNFR1- and TNFR2-dependent manner [44, 72] which resulted in increased intestinal permeability* in vivo* [44]. TNF signalling upregulated expression of inducible nitric oxide synthase (iNOS) which led to enhanced expression of a proapoptotic protein p53 [72]. On the other hand, TNF participates also in transactivation of epidermal growth factor receptor (EGFR) [75] which signaling upregulates the expression of cyclooxygenase-2 (COX-2) [76]. Increased expression of COX-2 has been associated with enhanced cell resistance to apoptosis, inflammation, and promotion of tumour progression [77]; therefore this aspect of TNF activity might have relevance to development of IBD-associated cancers of the GI tract [78].

### 3.2. Anti-TNF Agents in IBD Therapy

Currently, IBD therapy based on blocking biological activity of TNF involves the use of the following anti-TNF agents approved by Food and Drug Administration (FDA) and European Medicines Agency (EMA): (1) infliximab: chimeric monoclonal anti-TNF antibody (approved by FDA and EMA for treatment of CD and UC); (2) adalimumab: human monoclonal anti-TNF antibody (approved by FDA for treatment of CD in adults and by EMA for treatment of CD and UC); (3) certolizumab pegol: humanized Fab' fragment of anti-TNF antibody conjugated to polyethylene glycol (approved by FDA only for treatment of CD) [79]; (4) golimumab: human monoclonal anti-TNF antibody (approved by FDA and EMA for treatment of UC) [80]. Infliximab, adalimumab [81], and certolizumab pegol [82] are effective in the treatment of patients with moderate and severe CD who do not respond to standard anti-inflammatory drugs and also when used as a first-line therapy in CD. Moreover, randomised, controlled trials showed also that infliximab [83, 84], adalimumab [85, 86], and golimumab [87, 88] induced remission in steroid- or immunosuppressant-refractory patients with moderate or severe UC. However, 10 to 40% of CD patients (depending on selection criteria) and up to 50% of UC patients do not respond to anti-TNF therapy (primary resistance) and about one-third become resistant (secondary loss of response) at 12 months after initiation of anti-TNF treatment [89–91]. Interestingly, switching to another anti-TNF agent is effective in over 50% of nonresponsive patients [92, 93]. Failure to respond to anti-TNF therapy may result from pharmacokinetics of drugs, development of antibodies against the drugs, or activity of other, TNF-independent, proinflammatory pathways in IBD patients [91, 94, 95].

It has also to be noted that blockade of TNF biological activity in IBD therapy may result in several adverse side effects [82], including acute or delayed hypersensitivity reactions to anti-TNF agents [96, 97], elevated risk of bacterial, mycobacterial, viral, and fungal infections [98] (although meta-analysis of clinical trials did not show increased rate of infections in the course of anti-TNF treatment [89]), or neurological complications [99–101]. Combinatory therapy of CD patients with glucocorticoids, immunomodulators, and TNF inhibitors may be associated with an increased risk of non-Hodgkin's lymphoma, lung, skin, and other types of cancers, although no causative relationship of anti-TNF antibodies and carcinogenesis has been proven [90]. Anti-TNF therapy may lead also to paradoxical inflammatory skin (eczema and psoriasis) and joint (polyarthralgia) or ocular (uveitis and scleritis) manifestations [102]. Other paradoxical reactions include also demyelinating central nervous system disorders, sarcoidosis, development of anti-nuclear antibodies, and, in rare cases, lupus [89]. Mechanisms leading to these paradoxical reactions are not currently well known and most probably involve multiple pathogenic pathways. For example, it has been recently reported that psoriasiform skin lesions characterised by Th17 and Th1 cell infiltrates developed in nearly 5% of anti-TNF-treated patients with IBD and that smoking was identified as a main risk factor. Interestingly, anti-IL-12/IL-23 antibody treatment was found to be a highly effective therapy for these lesions [103].

Despite high efficacy of the majority of anti-TNF antibodies in the therapy of a considerable proportion of both CD and UC patients [90], the precise mechanisms of action underlying the efficacy of anti-TNF agents in IBD therapy have not been fully explained. In the last decade multiple mechanisms of the anti-TNF antibodies such as blocking and neutralizing of TNF molecules, regulation of cell adhesion molecule expression, induction of regulatory macrophages, or direct induction of apoptosis of T lymphocytes and macrophages in the mucosal lamina propria and peripheral blood have been proposed [104, 105]. However, the results of newer studies suggest that increased apoptosis of Treg cells, an important subset of T lymphocytes, may play an important role in the pathogenesis of IBD and can be reversed by anti-TNF*α* treatment [106, 107]. Moreover, infliximab and adalimumab (but not etanercept and certolizumab) were shown to induce regulatory macrophages (CD206+) in an Fc region-dependent manner.* In vitro* these macrophages produced anti-inflammatory cytokines and inhibited proliferation of activated T cells [108], whereas* in vivo *a significant induction of regulatory macrophages was observed in IBD patients with mucosal healing after treatment with infliximab and this induction was absent in patients without mucosal healing response [109].

Recently, Leal et al. [110] using whole-genome transcriptional analysis have found that anti-TNF treatment reduced expression of a set of proinflammatory genes (including IL-6, IL-23p19, and MMP9) as well as genes of cell-activation markers (CD69, CD83, and VCAM-1) in patients who both did and did not respond to this kind of therapy, suggesting that it is not only the proinflammatory function of TNF that is targeted by anti-TNF therapy. Moreover, they identified IL1B and IL17A as genes that remained altered in nonresponders, which suggests that respective proteins or their signaling pathways may present a novel therapeutic target in IBD.

Since many studies have linked TNF to increased permeability of intestinal epithelium [63–65], it is highly possible that anti-TNF agents are involved in the protection of epithelial barrier. Indeed, administration of infliximab restored the proper function of intestinal epithelium in CD patients [42, 111] and prevented TNF-induced rearrangement of tight junction proteins (notably, occludin and zonula occludens-1) in dinitrobenzene sulfonic acid- (DNBS-) induced colitis in mice [112]. These findings have been supported by a recent* in vitro* study on intestinal epithelial cell lines Caco-2 and T84 which showed that adalimumab restored expression of tight junction proteins claudin-1, claudin-2, and claudin-3 downregulated by exposure to TNF and IFN-*γ* [113]. Other studies demonstrated that infliximab and adalimumab induced apoptosis of CD4(+) helper T cells expressing TNFR2 and macrophages isolated from colonic lamina propria of CD patients but not healthy subjects [23]. Furthermore, Eder et al. [114, 115] found that infliximab and adalimumab promoted apoptosis of intestinal lamina propria mononuclear cells present in inflamed but not noninflamed areas of CD patients' colonic mucosa via intrinsic pathway mediated by Bcl-2 family proteins. Thus, infliximab and adalimumab not only protect intestinal epithelial integrity but also may suppress inflammatory process by inducing apoptosis of immune cells present in intestinal mucosa.

The ongoing research aimed at the elucidation of the cellular and molecular mechanisms of the anti-inflammatory activity of some but not all anti-TNF antibodies in IBD should help in designing more target-effective biological drugs. Etanercept, a nonantibody soluble fusion protein composed of the extracellular domain of TNFR2 and the hinge and Fc fragments of human IgG1 antibody [116], is an anti-TNF agent approved by FDA and EMA for treatment of rheumatoid arthritis, psoriasis, psoriatic arthritis, ankylosing spondylitis, and juvenile idiopathic arthritis but not IBD. Even though experiments on mice with DNB-induced colitis showed that etanercept reduced levels of circulating TNF and prevented apoptosis of enterocytes equally well as infliximab [112], studies on humans proved that response rates to etanercept in CD treatment were comparable to placebo [117]. A study by Scallon et al. [116] showed that infliximab binds both monomeric and trimeric forms of soluble and transmembrane TNF, whereas etanercept forms only unstable complexes with soluble TNF which may contribute to prolonged half-life of circulating TNF. Furthermore, in contrast to infliximab, etanercept did not induce apoptosis of activated T cells isolated from CD patients and healthy control subjects [104]. The failure of etanercept in IBD therapy can be attributed to its inability, in contrast to infliximab and adalimumab, to inhibit T cell proliferation and to induce regulatory macrophages [108], caused probably by differences in infliximab and etanercept binding to TNF.

## 4. TL1A

TL1A (TNF-like molecule 1A; TNFSF15) is the most recently discovered member of the TNF superfamily, identified for the first time in 2002 [118]. In humans, there are three different isoforms of the protein generated from TNFSF15gene as a result of alternative splicing: VEGI-174 (174 amino acids), VEGI-192 (192 amino acids), and the full-length product, TL1A (VEGI-252; 252 amino acids) [118–120], although VEGI-174 is most probably a cloning artefact [118]. Primary function of VEGI-192 is the inhibition of angiogenesis [121], whereas TL1A is a proinflammatory factor involved in the pathogenesis of several autoimmune diseases, including arthritis, allergic lung inflammation, autoimmune encephalomyelitis, and inflammatory bowel disease [26, 122–124].

Similarly to TNF, TL1A exists in a soluble or transmembrane form [125–128]. It has been shown that recombinant human TL1A forms a homotrimer resembling the trimeric structure of other TNF superfamily members [129]; however, still very little is known about the quaternary structure of the native form of TL1A molecule.

TL1A expression is primarily found on activated cells of the immune system, such as dendritic cells, macrophages [128], and CD4(+) and CD8(+) T cells [122, 123], whereas very little TL1A was found on nonactivated immune cells [118]. Known inducers of TL1A expression are TNF, IL-1 [118], Fc fragments of IgG1 antibodies [128], and certain parasite- or bacteria-related toll-like receptors (TLR) ligands, including synthetic bacterial lipoprotein Pam3CSK4 (ligand for TLR1 and TLR2), lipopeptide FSL (ligand for TLR2), polyinosinic-polycytidylic acid (ligand for TLR3), LPS (ligand for TLR4), single-stranded RNA (ligand for TLR7), unmethylated DNA sequences (ligands for TLR9), and tachyzoite antigen (ligand for TLR11) [123, 130].

The main receptor for TL1A is death receptor 3 (DR3; TNFRSF25) [118], structurally similar to TNFR1 [131]. DR3 was found to be strongly upregulated on activated monocytes [132], NK cells [133], NKT cells [122], and B cells [134], as well as CD4(+) T helper and CD8(+) T cytotoxic cells [133, 135]. TL1A, similarly to FasL and LIGHT, binds also soluble decoy receptor 3 (DcR3, TNFRSF6B) which prevents functional TL1A/DR3 signalling [127, 136].

Even though DR3 contains the death domain, TL1A has been shown to induce apoptosis only in the erythroleukaemic cell line TF-1 treated with an inhibitor of protein synthesis, cycloheximide (CHX) [118, 137]. In activated T cells, however, TL1A did not induce apoptosis even in the presence of CHX [118]. Instead, TL1A/DR3 interactions in lymphocytes triggered proliferative and costimulatory signals through activation of NF-*κ*B-mediated pathways [123, 133]. Thus, TL1A is a proinflammatory molecule which primarily costimulates proliferation and effector functions of CD8(+) cytotoxic T cells [138] as well as Th1, Th2, and Th17 [30, 123, 126, 139, 140] cells in the presence of TCR stimulation; however, in physiological conditions, TL1A is not required for the differentiation of these lymphocytes [123]. Furthermore, TL1A promotes also maturation of dendritic cells [141, 142] and production of proinflammatory cytokines (TNF, IL-8, and monocyte chemotactic protein 1, MCP-1) by macrophages [132]. Apart from conventional CD4(+) and CD8(+) T cells, TL1A/DR3 interaction promotes also proliferation of regulatory T cells (Tregs) [143, 144], although sustained TL1A stimulation* in vitro* dampens suppressive activity of Tregs [143–145]. Interestingly,* in vitro* studies also showed that TL1A inhibited differentiation of Tregs from their precursor cells [143, 145].

Certain alleles of TNFSF15gene which encode TL1A are associated with enhanced activity of TNFSF15promoter region and are considered to increase susceptibility to Crohn's disease [146]. TL1A protein and mRNA were upregulated in IBD and their synthesis was localized in CD patients to lamina propria infiltrating cells such as macrophages, dendritic cells, and CD4(+) and CD8(+) T cells [139, 140, 147] as well as plasma cells isolated from colon mucosa of UC patients [125]. IBD patients had also a higher proportion of DR3-expressing lamina propria T cells than healthy subjects [125, 126] and the amount of TL1A protein as well as the number of TL1A-positive cells correlated positively with the severity of inflammation, most significantly in CD [125]. Furthermore, studies on transgenic mice showed that constitutive elevated expression of TL1A on T cells or dendritic cells resulted in enhanced T cell activation and upregulation of IL-13, IL-17A, and IFN-*γ* mRNA levels in intestinal mucosa and mesenteric lymph nodes as well as spontaneous development of bowel inflammation [143, 144].

Recently, a population of CD161(+)CD4(+) T cells has been identified as a primary target of TL1A in IBD [148], although other subpopulations of T cells may also respond to TL1A costimulation. CD161(+)CD4(+) T cells express DR3 [149] and their gut tropism is established by high expression of intestine-homing molecules such as integrin *β*7 and chemokine CCR6 [149, 150]. They bear characteristics of Th17 cells and have been shown to produce proinflammatory cytokines IL-17, IL-22, and IL-13. In inflammatory conditions, however, they may revert their phenotype to Th1 type and produce IFN-*γ* [149–151]. In synergy with other proinflammatory cytokines, such as IL-12 and IL-18 or IL-23, TL1A further enhances the inflammatory process by increasing production of IFN-*γ*, IL-8, and IL-6 by lymphocytes [30, 125, 139, 140]. Thus, TL1A involvement in IBD pathomechanisms may result from enhanced costimulation of effector T cells and local upregulation of proinflammatory cytokines production in parallel to defective generation of peripheral Tregs and inhibition of suppressive activity of preexisting Tregs [45, 46].

As one of the key regulators of inflammatory pathways, TL1A appears to be a promising therapeutic target for patients with T cell-mediated autoimmune diseases, including IBD, although to this day none of TL1A blocking agents has yet been tested in clinical trials. There are, however, reports showing that antibody-mediated inhibition of TL1A biological activity prevents the development of dextran sodium sulphate- (DSS-) induced and T cell transfer-induced experimental bowel inflammation in mice [139].

## 5. FasL

Fas ligand (FasL, CD95L, and TNFSF6) and its receptor Fas (CD95, TNFRSF6) are other members of the TNF superfamily involved in the pathogenesis of IBD. Cytotoxic T cells and natural killer (NK) cells use FasL to kill tumour cells or viruses-infected cells which express Fas. FasL is involved also in maintaining immune homeostasis and preventing autoimmunity via a mechanism known as activation-induced cell death (AICD) which relies on killing activated T cells following their expansion and differentiation in a FasL-/Fas-dependent manner, thus preventing hyperactivation of T cell-mediated immunity [47, 152].

FasL is a transmembrane molecule, although it can be enzymatically cleaved from cells [153]. An* in vivo* mouse study showed, however, that only the transmembrane, but not soluble, FasL was capable of triggering cell death [154]. In contrast to Fas which is constitutively or inducibly expressed on many different cell types, including colon epithelial cells [155], FasL expression is tightly regulated and limited to activated CD4(+) and CD8(+) T cells, NK cells, and monocytes [47]. In physiological conditions, Paneth cells are the only cells of the intestinal epithelium which express FasL [156]. Expression of FasL was found also in tissues and organs that lack resident or infiltrating lymphocytes (e.g., eye, trophoblast, or testis) and on neurons and astrocytes as well as in several tumours where it may contribute to the suppression of local immune responses* via* induction of T cell apoptosis [47].

The majority of studies concerning the role of FasL and Fas in IBD have been conducted in patients with ulcerative colitis rather than Crohn's disease. Expression of FasL was significantly elevated on CD3(+) lymphocytes infiltrating colonic lamina propria in patients with active UC but not in UC remission, active or remission CD, or healthy subjects [34, 35]. Furthermore, serum concentration of systemic soluble Fas was lower in patients with active UC compared to healthy controls [35]. Nevertheless, there are also studies which showed upregulation of FasL in colonic lamina propria and intraepithelial lymphocytes of CD patients' mucosa [36].

The exact role of Fas/FasL system in IBD has not been fully elucidated. Taking into account the primary, proapoptotic function of Fas/FasL signalling, its possible role in IBD initially appeared to be similar to TNF/TNFR1 signalling: intestinal epithelial cells expressing Fas targeted by FasL(+) lymphocytes undergo apoptosis which may lead to the increased permeability of intestinal epithelium [157]. Indeed, an* in vitro* study demonstrated that ligation of Fas resulted in apoptotic death of intestinal epithelial cells isolated from mucosa of UC patients [158]. This concept was supported also by the fact that in healthy colon expression of FasL was restricted only to few mononuclear cells of lamina propria, suggesting that proapoptotic function of Fas/FasL system was not involved in regeneration of colonic epithelium but in pathogenesis of IBD [159]. A more recent study, however, showed that colonocytes isolated from patients with active UC had attenuated response to Fas-mediated apoptosis induction compared to healthy subjects and patients in remission [160]. Furthermore, authors of two mouse studies demonstrated that Fas-deficient mice were hypersensitive to dextran sodium sulphate- (DSS-) induced colitis [161] and did not show any significant reduction in tissue damage, even though they exhibited an increased rate of intestinal epithelial cell apoptosis in gut inflammation model based on administration of T cell activating anti-CD3 antibody [162]. These findings suggest that colonocytes may activate cytoprotective programs in response to inflammation and may not be oversensitive to Fas-dependent apoptosis as had been initially proposed [160].

Several studies showed that T cells from inflamed mucosa of CD and UC patients were more resistant to Fas-mediated apoptosis than control T cells from healthy individuals [163–165]. Suzuki et al. [166] found that in UC mucosa the population of CD45RO(+)CD4(+) T cells was less prone to Fas-mediated cell death than the population of CD45RO(+)CD8(+) T cells. Thus, potentially harmful, proinflammatory T cells may accumulate in the intestinal mucosa of IBD patients and induce tissue damage.

Fas and FasL, while playing an important role in the regulation of apoptosis, have also nonapoptotic functions. Fas contains the death domain and, in contrast to TNFR1, had been thought to be involved only in proapoptotic but not prosurvival signalling [167]. However, it has been shown recently that although strong Fas stimulation blocked activation of human CD4(+) helper T cells, weak Fas stimulation together with TCR signalling augmented their proliferation via activation of MAP kinases, transcription factors, and cell cycle activators [168].

FasL contributes to costimulation of T cells also by a phenomenon termed “reverse signalling.” Under this condition ligation of transmembrane FasL by functional Fas or DcR3 (a soluble decoy receptor for FasL, TL1A, and LIGHT) triggers signal transduction from FasL, resulting in the enhanced proliferation of mouse CD8(+) cytotoxic T cells [169–172]. These findings add much more complexity to possible roles of Fas/FasL system in the pathomechanisms of IBD which, theoretically, can be involved not only in direct disruption of epithelial continuity but also in costimulation of proinflammatory T cells. Since the details of Fas/FasL role in IBD still remain largely unknown, agents directly interfering with Fas signalling have yet not been tested for IBD treatment.

## 6. LIGHT 

LIGHT (lymphotoxin-like inducible protein that competes with glycoprotein D for binding herpesvirus entry mediator on T cell, TNFSF14), ligand for the lymphotoxin beta receptor (LT*β*R, TNFRSF3), and the herpesvirus entry mediator (HVEM; TNFRSF14) are expressed mostly on activated T cells, although they were found also on monocytes, granulocytes, and immature dendritic cells [173, 174]. LT*β*R and HVEM receptors do not contain the death domain; therefore they are considered to be involved in prosurvival signalling [167]. Indeed, interaction between LIGHT and HVEM was found to enhance proliferation and effector functions of CD8(+) cytotoxic T cells [25], stimulate expansion of CD4(+) helper T cells, and promote their differentiation into Th1 cells [28].

Several studies indicate that LIGHT contributes to the development of intestinal inflammation. Transgenic mice with elevated expression of LIGHT spontaneously develop colitis [175]. Adoptive transfer of mesenteric lymph node cells expressing LIGHT into immunodeficient RAG−/− mice resulted in Th1-mediated intestinal inflammation dependent on both LIGHT receptors (LT*β*R and HVEM) [49]. Furthermore, induction of colitis in mice by DSS resulted in strong upregulation of LIGHT mRNA in colon mucosa, whereas LIGHT-deficient mice showed significantly reduced symptoms of DSS-induced colon inflammation [176].

The pathological role of LIGHT in human IBD has been hardly investigated, although in IBD patients upregulation of LIGHT mRNA in inflamed intestinal mucosa when compared to noninflamed areas has been demonstrated [37]. Blockade of LIGHT as a way of IBD treatment has not been tested in clinical settings, although administration of anti-LIGHT antibodies reduced symptoms of DSS-induced colon inflammation in mice [176].

## 7. DcR3

DcR3, soluble receptor for TL1A, FasL, and LIGHT, is a member of the TNF receptor superfamily that does not contain the transmembrane domain [127, 136]. As a soluble receptor, it inhibits the interaction between its ligands and their membrane-bound receptors, thus suppressing their biological activity. Elevated expression of DcR3 was detected in inflamed mucosa and serum of CD and UC patients [177–179]. The biological significance of this phenomenon remains unclear, although DcR3 is thought to play a protective role in IBD. For example, Funke et al. [179] showed that DcR3, acting as a soluble decoy receptor, limited the bioavailability of FasL and protected intestinal epithelial cells from FasL-mediated apoptosis. In a similar way, DcR3 may also prevent the proinflammatory effect exerted by TL1A; thus, it has been proposed that upregulation of DcR3 expression during intestinal inflammation may have a compensatory, protective effect [45].

## 8. TRAIL

Certain reports indicate that another member of the TNF superfamily, TRAIL (TNF-related apoptosis inducing ligand, TNFSF10), expressed in a large variety of tissues including intestines may be involved in the pathogenesis of IBD [159]. Similarly to TNF, TRAIL is able to induce apoptosis and can also activate the prosurvival transcription factor NF-*κ*B. Five receptors of TRAIL have been identified to date. TRAIL-R1 (TNFRSF10A; DR4) and TRAIL-R2 (TNFRSF-10B; DR5) contain the death domain in their cytoplasmic fragments and are involved in functional TRAIL signalling [128]. Other molecules, TRAIL-R3 (TNFRSF10C; DcR1) without the death domain and TRAIL-R4 (TNFRSF10D; DcR2) with defective death domain and soluble osteoprotegerin (OPG, TNFRSF11B), are considered to be decoy receptors [167].

Expression of TRAIL was found to be downregulated in intestinal epithelial cells of IBD patients [180]; however, it was significantly elevated in mononuclear cells of the resected inflamed mucosa in both CD and UC patients with highly active, steroid-refractory disease [38, 180].

Even though the exact role of TRAIL in IBD pathogenesis remains undefined, the available data suggest that TRAIL-expressing mononuclear cells present in lamina propria disrupt the integrity of intestinal epithelium by inducing apoptosis of enterocytes. This notion was supported by an* ex vivo* study on ileal organ cultures which revealed that under inflammatory conditions TRAIL became a potent inducer of apoptosis in intestinal epithelial cells [38]. TRAIL is also a potent mediator of apoptotic death of intestinal fibroblasts in fibrostenosing intestinal areas in CD. Since collagen deposits and fibroblast proliferation are factors contributing to the development of strictures and fistulas, relatively common in CD patients, TRAIL can be involved also in tissue remodelling associated with CD [50].

## 9. TWEAK

TWEAK (TNF-like weak inducer of apoptosis, TNFSF12) acts through its receptor Fn14 (TNFRSF12; TWEAK-R) and has multiple biological activities, including stimulation of cell growth, induction of proinflammatory cytokines, and, in certain experimental settings, induction of apoptosis. TWEAK protein is expressed mostly in immune cells such as T cells, macrophages, or dendritic cells, although it was found also in nonhematopoietic cell types like astrocytes or endothelial cells [181]. Expression of TWEAK receptor, Fn14, was found on a variety of cells, including cells of intestinal mucosa. Fn14 does not contain the death domain and its stimulation with TWEAK results in activation of the transcription factor NF-*κ*B [181, 182].

There are very few studies concerning the role of TWEAK in IBD pathogenesis; however in the intestinal mucosa of UC patients messenger RNA levels of IL-13, TWEAK, and Fn14 increased with disease activity [39]. TWEAK deficiency or reduction of its biological activity by anti-TWEAK monoclonal antibodies reduced expression of proinflammatory cytokines, neutrophil, and macrophage infiltration decreasing severity of trinitrobenzenesulfonic (TNBS) acid-induced colitis in mice [51]. Furthermore, even though TWEAK alone did not induce damage or apoptosis of intestinal epithelial cells, it was required, together with Fn14 and TNF, for IL-13-induced activation of caspase-3 in enterocytes isolated from *γ*-irradiated mice [39].* In vitro *studies on rhabdomyosarcoma cell line Kym-1 showed that TWEAK upregulated expression of transmembrane TNF which in turn induced cell apoptosis via TNFR1 [183]. Furthermore, TWEAK stimulation synergistically enhanced TNFR1-mediated apoptotic cell death of Kym-1 cells [183] which can be explained by the fact that TWEAK induces translocation of antiapoptotic adaptor protein TRAF2 from the TNFR1 signalling complex, enhancing proapoptotic signalling of this receptor [184]. The question whether these mechanisms are active also* in vivo* in intestinal epithelium has not yet been studied.

## 10. Conclusions

**Figure 1:**
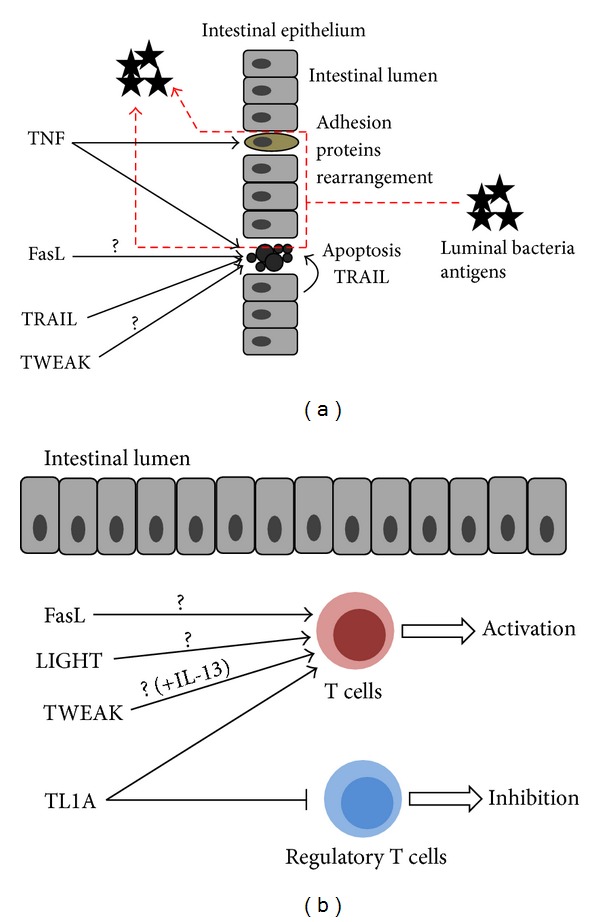
Two major mechanisms which implicate the molecules belonging to the TNFSF in the pathomechanisms of IBD. (a) Disruption of intestinal epithelium integrity allows luminal bacterial antigens to cross the epithelial barrier and migrate into the intestinal mucosa where they elicit immune responses. (b) Activation of mucosa-infiltrating T lymphocytes.

Members of the TNF superfamily contribute to the pathogenesis of IBD in two ways. (i) They disrupt the integrity of intestinal epithelium by altering the arrangement of adhesion proteins in enterocytes (TNF), inducing apoptotic death of enterocytes (TNF, FasL, TRAIL, and TWEAK), and/or (ii) they promote the proinflammatory activity of mucosa-infiltrating mononuclear cells (TNF, TL1A, LIGHT, TWEAK, and possibly FasL) and affect the activity of regulatory T cells and regulatory macrophages (Figure 1).

TNF superfamily members have attracted large attention as potential therapeutic targets in IBD treatment. Currently, however, the only TNFSF member targeted in clinical treatment of IBD is TNF. Another promising target, although still not tested in clinical trials, is TL1A which appears to be one of the key factors regulating the inflammatory pathways in IBD. The other members of TNF superfamily involved in IBD pathogenesis (FasL, LIGHT, TRAIL, and TWEAK) still require more in-depth studies to clearly define their function in intestinal inflammation. It has to be emphasized, however, that inflammatory injury of intestinal mucosa, a major feature of IBD, is mediated not only by the cross talk between various TNF superfamily members and their respective receptors since it results from the interactions of many cell types and inflammatory mediators which trigger multiple intracellular signalling pathways. Therefore, despite great therapeutic progress achieved in the treatment of Crohn's disease and ulcerative colitis by targeting TNF with various types of antibodies, further detailed studies are necessary to better understand the pathomechanisms of tissue injury in IBD aimed at defining more specific therapeutic targets.

## Figures and Tables

**Table 1:** Overview of the expression and function of the major members of TNSF superfamily in IBD.

TNFSF member and its expression	Receptors and their expression	Role in IBD pathogenesis	References
TNF—macrophages, NK cells, T cells, and B cells	(i) TNFR1—intestinal epithelial cells (ii) TNFR2—intestinal epithelial cells	Disruption of intestinal epithelium integrity by induction of adhesion proteins rearrangement and induction of intestinal cells apoptosis	[20, 43, 44]

TL1A—antigen-presenting cells and T cells	(i) DR3—T cells, NK cells, NKT cells, and regulatory T cells (ii) DcR3 (decoy)—activated T cells	Promotion of proinflammatory activity of T cells and inhibition of suppressive activity of regulatory T cells	[45, 46]

FasL—T cells, NK cells, monocytes, and Paneth cells	(i) Fas—intestinal epithelial cells and T cells (ii) DcR3 (decoy)—activated T cells	Possible disruption of intestinal epithelium integrity by induction of epithelial cells apoptosis. Possible involvement in accumulation of proinflammatory T cells in intestinal lamina propria	[20, 47, 48]

LIGHT—T cells, monocytes, granulocytes, and dendritic cells	(i) HVEM—T cells, B cells, and monocytes (ii) LT*β*R—nonlymphoid hematopoietic cells and stromal cells (iii) DcR3 (decoy)—activated T cells	Possible promotion of proinflammatory activity of Th1 cells	[20, 49]

TRAIL—intestinal epithelium, T cells, NK cells, and dendritic cells	(i) TRAIL-R1—almost all cell types (ii) TRAIL-R2—almost all cell types (iii) TRAIL-R3 (decoy)—almost all cell types (iv) TRAIL-R4 (decoy)—almost all cell types (v) OPG (decoy)—osteoclasts' precursors, endothelial cells, and other cell types	Disruption of intestinal epithelium integrity by induction of epithelial cells apoptosis. Possible contribution to development of fistulas and strictures in CD patients	[20, 38, 50]

TWEAK—T cells, macrophages, and dendritic cells	Fn14—intestinal mucosa and fibroblasts	Possible upregulation of proinflammatory cytokines and infiltration of lamina propria by inflammatory cells. Induction of intestinal cells apoptosis in cooperation with IL-13	[20, 39, 51]

**Table 2:** Biological effects of TNF exerted on intestinal epithelium.

TNF function	Model	References
(1) Rearrangement of cytoskeletal elements		
(i) Downregulation of zonula occludens-1 expression and alteration of its intracellular localization	(i) Caco-2 cells (*in vitro*) (ii) HT29-C1.16E cells (*in vitro*)	[63, 64]
(ii) Upregulation of myosin light chain kinase expression	(i) Caco-2 cells (*in vitro*) (ii) Mouse model (*in vivo*)	[65, 70]
(iii) Redistribution of zonula occludens-1, occludins, claudins, E-cadherins, and myosin light chain kinase to basolateral membranes of intestinal cells	Mouse model (*in vivo*)	[71]

(2) Induction of intestinal epithelial cells' apoptosis		
(i) Induction of intestinal cells' apoptosis via activation of caspase-3	Mouse model (*in vivo*)	[71]
(ii) Induction of intestinal epithelial cells' apoptosis via upregulation of iNOS and p53	Mouse model (*in vivo*)	[44, 72]

## Abbreviations

AICD: Activation-induced cell death
AP-1: Activator protein 1
CD: Crohn's disease
CHX: Cycloheximide
DcR3: Soluble decoy receptor 3
DD: Death domain
DNBS: Dinitrobenzenesulfonic acid
DR3: Death receptor 3
DSS: Dextran sodium sulphate
EGFR: Epidermal growth factor receptor
EMA: European Medicines Agency
FADD: Fas-associated death domain
FDA: Food and Drug Administration
GI: Gastrointestinal
IBD: Inflammatory bowel disease
IFN-*γ*: Interferon gamma
IL: Interleukin
iNOS: Inducible nitric oxide synthase
LIGHT: Lymphotoxin-like inducible protein that competes with glycoprotein D for binding herpesvirus entry mediator on T cell
LPS: Lipopolysaccharide
MCP-1: Monocyte chemotactic protein 1
MLC: Myosin light chain
MLCK: Myosin light chain kinase
MMP: Matrix metalloproteinase
mRNA: Messenger RNA
NF-*κ*B: Nuclear factor kappa B
NK: Natural killer
PBMCs: Peripheral blood mononuclear cells
TJ: Tight junction
TL1A: TNF-like protein 1A
TLR: Toll-like receptor
TNBS: Trinitrobenzenesulfonic acid
TNF: Tumour necrosis factor
TNFR1: TNF receptor 1
TNFR2: TNF receptor 2
TNFSF: Tumour necrosis factor superfamily
TRADD: TNF receptor-associated death domain
TRAF: Tumour necrosis factor receptor-associated protein
TRAIL: TNF-related apoptosis inducing ligand
TWEAK: TNF-like weak inducer of apoptosis
UC: Ulcerative colitis
VEGI: Vascular endothelial growth inhibitor.
